# Irisin evokes bradycardia by activating cardiac-projecting neurons of nucleus ambiguus

**DOI:** 10.14814/phy2.12419

**Published:** 2015-06-02

**Authors:** Eugen Brailoiu, Elena Deliu, Romeo A Sporici, G Cristina Brailoiu

**Affiliations:** 1 Center for Substance Abuse Research, Temple University School of MedicinePhiladelphia, Pennsylvania; 2 Department of Internal Medicine, Brandywine HospitalCoatesville, Pennsylvania; 3 Department of Pharmaceutical Sciences, Jefferson School of Pharmacy, Thomas Jefferson UniversityPhiladelphia, Pennsylvania

**Keywords:** Autonomic cardiovascular regulation, cytosolic Ca^2+^, membrane potential, vagal tone

## Abstract

Irisin is a newly identified hormone induced in muscle and adipose tissues by physical activity. This protein and its encoding gene have been identified in the brain; in addition, the precursor for irisin, FNDC5, can cross the blood-brain barrier. The fact that irisin is secreted during exercise together with the lower resting heart rate in athletes prompted us to investigate the effect of irisin on cardiac-projecting vagal neurons of nucleus ambiguus, a key regulatory site of heart rate. In vitro experiments in cultured nucleus ambiguus neurons indicate that irisin activates these neurons, inducing an increase in cytosolic Ca^2+^ concentration and neuronal depolarization. In vivo microinjection of irisin into the nucleus ambiguus promotes bradycardia in conscious rats. Our study is the first to report the effects of irisin on the neurons controlling the cardiac vagal tone and to link a myokine to a cardioprotective role, by modulating central cardiovascular regulation.

## Introduction

Irisin is a recently described myokine, secreted in the rodent and human skeletal muscle as a result of exercise (Bostrom et al. 2012). In muscle, exercise promotes the formation of fibronectin type III domain containing 5 (FNDC5, aka PeP, and FRCP2) and its cleavage to irisin by the transcriptional co-activator PPAR-*γ* co-activator-1 *α* (PGC1*α*) (Bostrom et al. 2012). Irisin stimulates thermogenesis and browning of white adipose tissue and increases energy expenditure (Bostrom et al. 2012). Irisin was found to be secreted also by the white adipose tissue and considered an adipokine that may contribute to a muscle-adipose tissue regulatory mechanism (Roca-Rivada et al. 2013; Crujeiras et al. 2015).

Besides skeletal muscle and adipose tissue, irisin has been recently identified in several other tissues including the brain (Aydin 2014; Aydin et al. 2014). Irisin was found in discrete regions of the brain and in the cerebrospinal fluid (Dun et al. 2013; Piya et al. 2014). FNDC5 brain expression is increased by exercise; in addition, the precursor for irisin, FNDC5, crosses the blood-brain barrier (Wrann et al. 2013), where it may be converted to irisin.

The circulating levels of irisin and the rodent-to-human translation of irisin biology have been subject of controversy (Crujeiras et al. 2015; Elsen et al. 2014; Sanchis-Gomar et al. 2014; Albrecht et al. 2015; Kerstholt et al. 2015). Clinical studies have shown increased circulating level of irisin after acute (Huh et al. 2012; Brenmoehl et al. 2014; Loffler et al. 2015) or chronic exercise (Bostrom et al. 2012; Ijiri et al. 2015). Increased serum irisin levels were found in active subjects (Moreno et al. 2015) or healthy centenarians, while young patients with myocardial infarction (Emanuele et al. 2014) or patients with type 2 diabetes (Xiang et al. 2014) had significantly reduced levels of irisin. Other studies found that, plasma irisin levels appear to be negatively correlated with high-density lipoproteins (HDL) cholesterol levels (Panagiotou et al. 2014), while positively correlated with markers of obesity (Stengel et al. 2013) and metabolic syndrome (Park et al. 2013), leading to the hypothesis that individuals at risk for cardiovascular disease may present with some type of irisin resistance (Park et al. 2013; Polyzos et al. 2013; Panagiotou et al. 2014).

Exercise has a beneficial effect on cardiovascular function, while sedentary lifestyle is a major risk factor for cardiovascular disease (Joyner and Green 2009; Warren et al. 2010). Regular physical activity is typically associated with lower resting heart rate, which is at least partially attributable to an increase in central cardiac vagal outflow (Melanson 2000; Buchheit et al. 2004; Sandercock et al. 2008; Joyner and Green 2009). The type and intensity of exercise determine the level of resting bradycardia and its mechanisms of control (Azevedo et al. 2014). The current study examined the effect of irisin on cardiac-projecting neurons of nucleus ambiguus, a critical site for central parasympathetic cardiac control (Mendelowitz 1999).

## Materials and Methods

### Ethical approval

Animal protocols were approved by the Institutional Animal Care and Use Committees of the Temple University and Thomas Jefferson University. All efforts were made to minimize the number of animals used and their suffering.

### Chemicals

Irisin recombinant (human, rat, mouse) was purchased from Phoenix Pharmaceuticals, Inc. (Burlingame, CA). In some experiments, irisin was heat inactivated by repeated (10 times) heating (75°C for 30 sec) and cooling (4°C for 1 min). All other chemicals were from Sigma-Aldrich (St. Louis, MO) unless otherwise mentioned.

### Animals

Sprague–Dawley rats (Charles River Laboratories, Wilmington, MA) were used in this study. Neonatal (1–2 days old) rats of either sex were used for retrograde tracing and neuronal culture and adult male rats (250–300 g) were used for the in vivo studies.

### Neuronal labeling and culture

Preganglionic cardiac vagal neurons of nucleus ambiguus were retrogradely labeled by intrapericardial injection of rhodamine [X-rhodamine-5-(and-6)-isothiocyanate; 5(6)-XRITC (Molecular Probes, Carlsbad, CA), as reported (Brailoiu et al. 2013, 2014). Medullary neurons were dissociated and cultured 24 h after rhodamine injection, as previously described (Brailoiu et al. 2013, 2014). In brief, the brains were quickly removed and immersed in ice-cold Hanks’ balanced salt solution (HBSS; Mediatech, Manassas, VA). The ventral side of the medulla (containing nucleus ambiguus) was dissected, minced, and the cells were subjected to enzymatic and mechanical dissociation. Cells were plated on glass coverslips in Neurobasal-A medium (Invitrogen, Life Technologies, Grand Island, NY) containing 1% GlutaMax (Invitrogen, Life Technologies), 2% antibiotic-antimycotic (Mediatech), and 10% fetal bovine serum. Cultures were maintained at 37°C in a humidified atmosphere with 5% CO_2_. Cytosine *β*-arabino furanoside (1 *μ*mol/L) was added to the culture to inhibit glial cell proliferation.

### Calcium imaging

Measurements of intracellular Ca^2+^ concentration, [Ca^2+^]_i_ were performed as previously described (Brailoiu et al. 2013, 2014). Briefly, cells were incubated with 5 *μ*mol/L Fura-2 AM (Invitrogen, Life Technologies) in HBSS at room temperature for 45 min, and washed with dye-free HBSS. Coverslips were mounted in an open bath chamber (RP-40LP, Warner Instruments, Hamden, CT) on the stage of an inverted microscope Nikon Eclipse TiE (Nikon Inc., Melville, NY), equipped with a Perfect Focus System and a Photometrics CoolSnap HQ2 CCD camera (Photometrics, Tucson, AZ). During the experiments, the Perfect Focus System was activated. Fura-2 AM fluorescence (emission 510 nm), following alternate excitation at 340 and 380 nm, was acquired at a frequency of 0.25 Hz. Images were acquired/analyzed using NIS-Elements AR software (Nikon). The ratio of the fluorescence signals (340/380 nm) was converted to Ca^2+^ concentrations (Grynkiewicz et al. 1985).

### Measurement of membrane potential

The relative changes of neuronal membrane potential were evaluated using bis-(1,3-dibutylbarbituric acid)-trimethine-oxonol, DiBAC_4_(3), a voltage-sensitive dye, as reported (Brailoiu et al. 2013, 2014). Cultured ambiguus neurons were incubated for 30 min in HBSS containing 0.5 mmol/L DiBAC_4_(3) and the fluorescence monitored at 0.17 Hz, excitation/emission: 480 nm/540 nm. Calibration of DiBAC_4_(3) fluorescence following background subtraction was performed using the Na^+^-K^+^ ionophore gramicidin in Na^+^-free physiological solution and various concentrations of K^+^ (to alter membrane potential) and N-methylglucamine (to maintain osmolarity).

### Surgical procedures

Adult male Sprague–Dawley rats were anesthetized with a mixture of ketamine hydrochloride (100–150 mg/kg) and acepromazine maleate (0.2 mg/kg) as reported (Brailoiu et al. 2013, 2014). Animals were placed into a stereotaxic instrument; a guide C315G cannula (PlasticsOne, Roanoke, VA) was bilaterally inserted into the nucleus ambiguus. The stereotaxic coordinates for identification of nucleus ambiguus were: 12.24 mm posterior to bregma, 2.1 mm from midline and 8.2 mm ventral to the dura mater (Praxinos and Watson 1998). A C315DC cannula dummy (PlasticsOne) was used to prevent contamination. For transmitters implantation, a 2-cm long incision was made along the linea alba. A calibrated transmitter (E-mitters, series 4000; Mini-Mitter, Sunriver, OR) was inserted in the intraperitoneal space, as previously described (Brailoiu et al. 2013, 2014). Subsequently, the abdominal musculature and dermis were sutured independently, and animals returned to individual cages.

### Telemetric heart rate monitoring

The signal generated by transmitters was collected via series 4000 receivers (Mini-Mitter, Sunriver, OR), as previously described (Brailoiu et al. 2013, 2014). VitalView™ software (Mini-Mitter, Sunriver, OR) was used for data acquisition. Each data point represents the average of heart rate per 30 sec.

### Noninvasive blood pressure measurement

In rats with cannula inserted into the nucleus ambiguus, blood pressure was noninvasively measured using a volume pressure recording sensor and an occlusion tail-cuff (CODA System, Kent Scientific, Torrington, CT), as described (Brailoiu et al. 2014). One week after the insertion of the cannula, rats were exposed to handling and training every day for 1 week. The maximum occlusion pressure was 200 mm Hg, minimum pressure 30 mm Hg and deflation time 10 sec. Two measurements were done per 30 sec (one cycle) and the average was used to calculate heart rate, systolic, diastolic and mean arterial blood pressure. Ten acclimatization cycles were done before starting the experiments.

### Microinjection into nucleus ambiguus

One week after surgery (telemetric studies), or after another week of training (tail-cuff measurements), the solution to be tested was bilaterally microinjected into the nucleus ambiguus, using the C315I internal cannula (33 gauge, PlasticsOne), without animal handling. In the tail-cuff method, trained rats were in the animal holder for the duration of the experiment. For recovery, at least 2 h were allowed between two injections. Injection of L-glutamate (5 mmol/L, 50 nL with Neuros Hamilton syringe, Model 7000.5 KH SYR) was used for the functional identification of nucleus ambiguus (Brailoiu et al. 2013, 2014). At the end of the experiments, the microinjection sites were identified, and compared with a standard rat brain atlas (Praxinos and Watson 1998) as previously described (Brailoiu et al. 2013).

### Statistical analysis

Data were expressed as mean ± standard error of mean. One-way ANOVA followed by post hoc analysis using Bonferroni and Tukey tests was used to evaluate significant differences between groups; *P* < 0.05 was considered statistically significant.

## Results

### Irisin elevates [Ca^2+^]_i_ in cardiac vagal neurons of nucleus ambiguus

**Figure 1:**
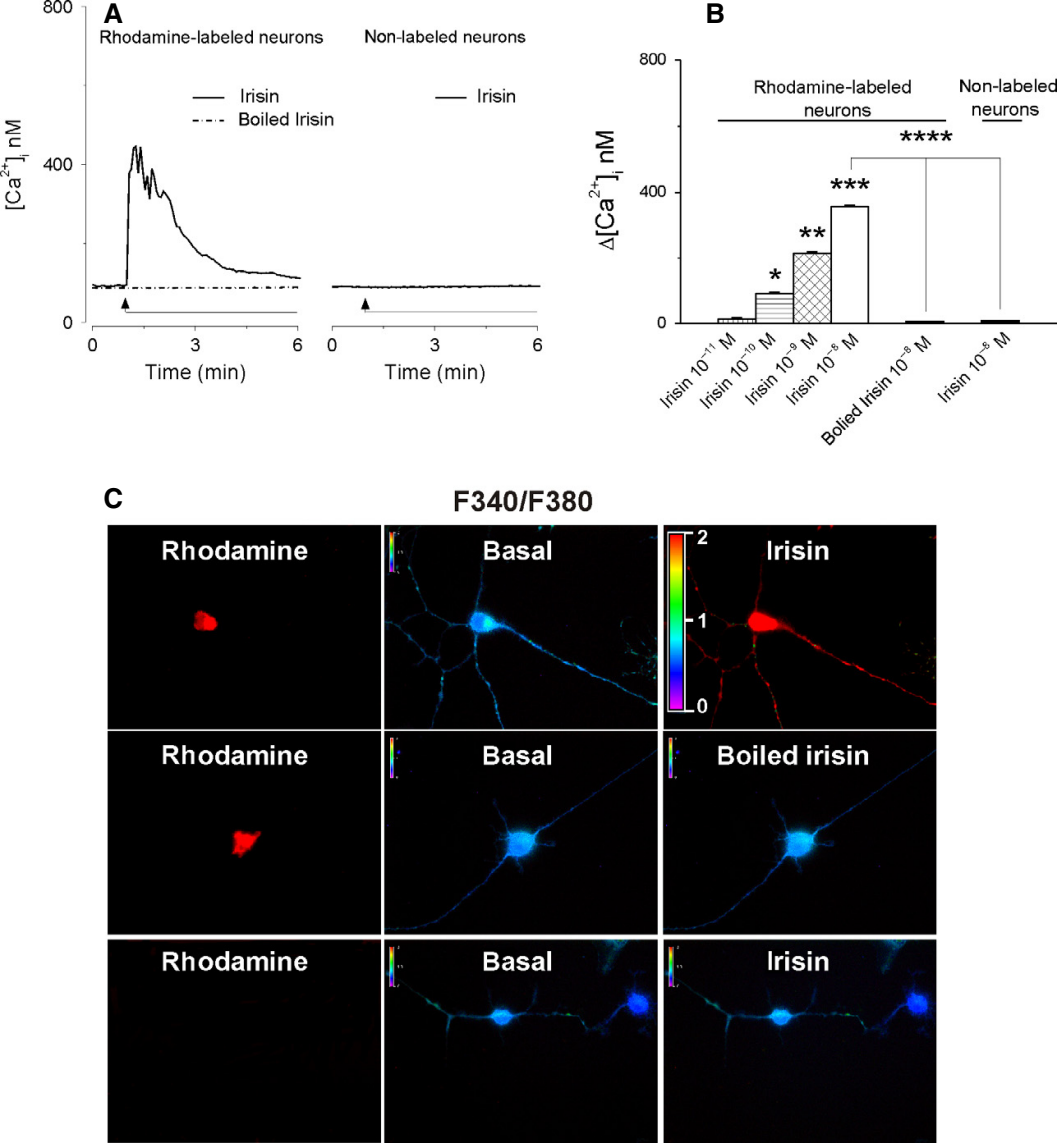
Irisin increases [Ca^2+^]_i_ in cardiac vagal neurons of nucleus ambiguus. (A) Representative recordings of the Ca^2+^ responses produced by irisin (10^−8^ mol/L), boiled irisin (10^−8^ mol/L) in rhodamine-labeled neurons, and by irisin (10^−8^ mol/L) in nonlabeled neurons. (B) Comparison of the mean amplitudes of the Ca^2+^ responses produced by increasing concentrations of irisin (10^−11^–10^−8^ mol/L) and by heat-inactivated irisin (10^−8^ mol/L) in rhodamine-labeled neurons and by irisin (10^−8^ mol/L) in nonlabeled neurons; *P *<* *0.05 as compared to basal [Ca^2+^]_i_, (*), to the increase in [Ca^2+^]_i_ produced by irisin (10^−10^ mol/L) (**), irisin 10^−9^ mol/L (***), and irisin 10^−8^ mol/L (****). (C) Changes in Fura-2 fluorescence ratio (340 nm/380 nm) of rhodamine-labeled neurons upon administration of 10^−8^ mol/L irisin (top) or 10^−8^ mol/L heat-inactivated irisin (middle), and of nonlabeled neurons upon administration of 10^−8^ mol/L irisin (bottom).

Application of irisin to rhodamine-labeled cardiac preganglionic neurons of nucleus ambiguus resulted in a fast and robust increase in [Ca^2+^]_i_, (Fig.1A); this response was absent in rhodamine-labeled neurons (cardiac-projecting) treated with boiled irisin as well as in neurons nonlabeled with rhodamine (presumably neighboring neurons) treated with irisin (Fig.1A). Increasing concentrations of irisin (10^−11^ mol/L, 10^−10^ mol/L, 10^−9^ mol/L and 10^−8 ^mol/L) produced an increase in [Ca^2+^]_i_ by 16 ± 1.8 nmol/L, 92 ± 2.7 nmol/L, 214 ± 3.8 nmol/L, and 357 ± 2.4 nmol/L (*n* = 6 neurons for each concentration), respectively (Fig.1B). With the exception of the lowest concentration tested (10^−11 ^mol/L), all the other concentrations of irisin (10^−10^–10^−8^ mol/L) produced a statistically significant response (*P* < 0.05). Boiled irisin increased [Ca^2+^]_i_ only by 8 ± 0.9 nmol/L (*n* = 6) (Fig.1B). Representative examples of fluorescence ratio 340/380 changes are shown in Fig.1C.

### Irisin depolarizes cardiac vagal neurons of nucleus ambiguus

**Figure 2:**
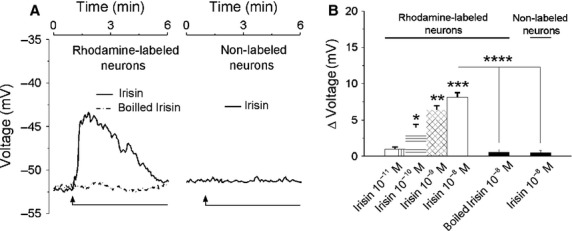
Irisin depolarizes cardiac vagal neurons of nucleus ambiguus. (A) Representative examples indicating changes in neuronal membrane potential produced by irisin (10^−8^ mol/L) and heat-inactivated (boiled) irisin (10^−8^ mol/L) in rhodamine-labeled neurons and by irisin (10–8 mol/L) in nonlabeled neurons (B) Concentration-dependent depolarizations produced by irisin (10^−11^–10^−8^ mol/L) and lack of effect of boiled irisin (10^−8^ mol/L) in rhodamine-labeled neurons; irisin (10^−8^ mol/L) did not affect the membrane potential in nonlabeled neurons; *P *<* *0.05 as compared to the resting membrane potential (*), to the response to irisin (10^−10^ mol/L) (**), irisin 10^−9^ mol/L (***), and irisin 10^−8^ mol/L (****).

Irisin produced the depolarization of cardiac-projecting parasympathetic neurons of nucleus ambiguus (Fig.2A), while boiled irisin did not affect the membrane potential (ΔVm was 0.58 ± 0.37 mV, *n* = 6 neurons; Fig.2A, B). Irisin (10^−8^mol/L) did not produce a depolarization of the cultured neurons that were nonlabeled with rhodamine (ΔVm was 0.47 ± 0.29 mV, *n* = 6 neurons Fig.2A, B). The mean amplitude of the depolarizations produced by irisin at 10^−11^ mol/L, 10^−10^ mol/L, 10^−9^ mol/L and 10^−8^mol/L was 0.96 ± 0.31 mV, 3.94 ± 0.46 mV, 6.37 ± 0.59 mV, and 8.11 ± 0.63 mV (*n* = 6 cells for each concentration; the effect was statistically significant (*P* < 0.05) for irisin 10^−10^–10^−8^ mol/L (Fig.2B).

### Microinjection of irisin into the nucleus ambiguus produces bradycardia in conscious rats

**Figure 3:**
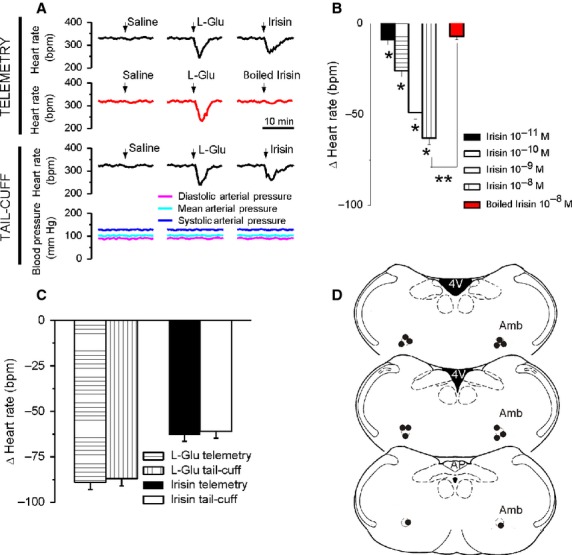
Microinjection of irisin into the nucleus ambiguus produces bradycardia in conscious rats. (A) Typical recordings of heart rate and blood pressure during microinjection of saline, L-glutamate (L-Glu, 5 mmol/L, 50 nL) and either irisin (10^−8^ mol/L, 50 nL) or boiled irisin (10^−8^ mol/L, 50 nL), obtained using the telemetric method (top traces) or the tail-cuff method (bottom traces). (B) Comparison of the bradycardic responses elicited by microinjection of irisin (10^−11^–10^−8^ mol/L) and by heat-inactivated irisin (10^−8^ mol/L); *P *<* *0.05 compared to the response to the other concentrations of irisin (*), or to the effect of irisin10^−8^ mol/L (**). (C) Consistency of heart rate monitoring using invasive (telemetry) or noninvasive (tail-cuff) methods is indicated by the similarity of the responses induced by either L-Glu or irisin in the two paradigms. (D) Illustration of microinjection sites (dark dots) on coronal medullary sections. Abbreviations: AP, area postrema; Amb, nucleus ambiguus; 4V, fourth ventricle.

In conscious, freely moving rats, bearing cannula implanted into the nucleus ambiguus, microinjection of control saline (50 nL) produced negligible effects on heart rate, monitored telemetrically (Fig.3A, top). The correct placement of the cannula into the nucleus ambiguus was indicated by the bradycardic effect produced by microinjection of L-glutamate (5 mmol/L, 50 nL), which was not associated with a blood pressure response (Fig.3A) as previously reported (Marchenko and Sapru 2003; Brailoiu et al. 2014).

Two hours after L-glutamate administration, microinjection of irisin (50 nL of either 10^−11^ mol/L, 10^−10^ mol/L, 10^−9^ mol/L or 10^−8^ mol/L) reduced the heart rate by 9 ± 2.6 beats per minute (bpm), 26 ± 3.2 bpm, 49 ± 3.7 bpm, and 63 ± 3.4 bpm (*N* = 5 rats for each concentration of irisin tested), respectively (Fig.3B). No effects on heart rate were observed upon administration of heat-inactivated irisin into the nucleus ambiguus of conscious rats (Δ heart rate = 7 ± 1.9 bpm, *n* = 5 rats, Fig.3A, B).

Cardiovascular monitoring using tail-cuff methods identified similar bradycardic effects (Fig.3A bottom) to those assessed by telemetry measurement, and absence of any effect on blood pressure (Fig.3A bottom). As previously reported (Brailoiu et al. 2014), we found a good correlation between these two methods: L-glutamate decreased the heart rate by 89 ± 3.9 bpm (telemetry) and by 87 ± 4.1 bpm (tail cuff), while the bradycardic responses to irisin measured 63 ± 3.4 bpm and 61 ± 3.7 bpm, respectively (*N* = 5 rats, Fig.3C). A diagram indicating the sites of microinjection is illustrated in Fig3D.

## Discussion

Endurance exercise training has beneficial effects on metabolism (Bostrom et al. 2012), brain cognitive functions (Cotman et al. 2007) and cardiovascular activity (Sandercock et al. 2008). An important mechanism underlying the resting sinus bradycardia induced by high-intensity aerobic exercise consists in elevated cardiac parasympathetic tone (Borresen and Lambert 2008; Sandercock et al. 2008; Azevedo et al. 2014). Since irisin is secreted by muscle and adipose tissue during exercise (Bostrom et al. 2012; Crujeiras et al. 2015), we investigated its possible role in modulating the cardiac vagal tone.

FNDC5 expression has been described in the rodent brain (Ferrer-Martinez et al. 2002; Teufel et al. 2002), and its secreted form, irisin, was detected by immunohistochemistry in several regions of rodent and human brain (Dun et al. 2013; Aydin et al. 2014; Piya et al. 2014). The presence of irisin immunoreactivity in nucleus ambiguus was not clearly identified, However, this may be due to the specificity of the commercial available antibodies to detect FNDC5/irisin which has been recently questioned (Albrecht et al. 2015). FDNC5 brain expression is increased by endurance exercise (Wrann et al. 2013), while circulating irisin levels were increased after acute (Huh et al. 2012; Brenmoehl et al. 2014; Loffler et al. 2015) or chronic exercise (Bostrom et al. 2012; Ijiri et al. 2015). In addition, peripheral delivery of FNDC5 with adenoviral vectors enhances central gene expression, suggesting that this effect is mediated by a secreted, circulating form of FNDC5 that crosses the blood-brain barrier (Wrann et al. 2013). A recent study (Piya et al. 2014), suggested a peripheral origin of the hypothalamic irisin. Further studies are needed to clarify the origin of the brain irisin, especially since high variability in the irisin levels determined in biological fluids with commercially available kits for ELISA, EIA, and RIA has been reported (Sanchis-Gomar et al. 2014; Albrecht et al. 2015).

Information regarding cellular effects of irisin is scarce. To date, it is known that irisin stimulates mitogen-activated protein kinases pathways in human umbilical vein endothelial cells (Song et al. 2014) and in white adipocytes (Zhang et al. 2014), eliciting proliferation and expression of browning-specific genes, respectively. With respect to the central nervous system, it has been found that exercise activates a PGC-1*α*/FNDC5/brain-derived neurotrophic factor in hippocampal neurons, but the contribution of irisin to this effect has not been established (Wrann et al. 2013).

Our results indicate that irisin activates cardiac-projecting neurons of nucleus ambiguus promoting a concentration-dependent increase in cytosolic Ca^2+^ concentration. This subset of neurons was identified by retrograde labeling with rhodamine. The pharmacological tools currently available for characterization of the effects of irisin are limited, as its receptor is not known and an antagonist is not yet available. As a result, we used inactivated irisin as a negative control, as well as the assessment of the effects of irisin on cultured brainstem neurons that were not labeled with rhodamine, presumably neighboring neurons.

Irisin produced the depolarization of rhodamine-labeled, cardiac-projecting vagal neurons of nucleus ambiguus, but not of those nonlabeled with rhodamine. Depolarization of cardiac-projecting neurons leads to release of acetylcholine to cardiac ganglia and subsequent bradycardia (Mendelowitz 1999). To validate this mechanism in our system, we carried out in vivo studies. Microinjection of irisin into the nucleus ambiguus elicited bradycardia in conscious rats. Our study is the first to show direct irisin-mediated responses in brainstem neurons that regulate the heart rate. Our findings, corroborated with previous reports indicating higher plasma levels of irisin after exercise (Huh et al. 2012; Loffler et al. 2015) or in active as compared to sedentary subjects (Moreno et al. 2015) suggest that irisin may be involved in a compensatory vagal activation. In addition, irisin may contribute to the increased cardiac parasympathetic tone reported in athletes (Borresen and Lambert 2008). To our knowledge, this is the first report to link a myokine to a cardioprotective role by modulating the central cardiovascular regulation.

## Conflict of Interest

None declared.
